# Exploring the Mechanism of Umami Peptide Binding with the T1R1/T1R3 Receptor via Molecular Dynamics Simulations

**DOI:** 10.3390/molecules31122125

**Published:** 2026-06-16

**Authors:** Chenyi Lu, Binghan Wu, Xianbing Xu, Haiyang Zhang

**Affiliations:** 1 Department of Biological Science and Engineering, School of Chemistry and Biological Engineering, University of Science and Technology Beijing, Beijing 100083, China; m202310916@xs.ustb.edu.cn (C.L.); m202321064@xs.ustb.edu.cn (B.W.); 2 National Engineering Research Center of Seafood, Collaborative Innovation Center of Seafood Deep Processing, School of Food Science and Technology, Dalian Polytechnic University, Dalian 116034, China; xianbingxu@dlpu.edu.cn

**Keywords:** umami peptide, T1R1/T1R3 taste receptor, molecular docking, molecular dynamics simulation

## Abstract

The Pacific oyster (*Crassostrea gigas*) is well known for its pronounced umami taste. Here the interaction between the T1R1/T1R3 taste receptor and three oyster-based peptides, namely, FLNQDEEAR (FR-9), EEFLK (EK-5), and FNKEE (FE-5), was investigated via molecular docking and molecular dynamics (MD) simulations, as well as molecular mechanics Poisson–Boltzmann surface area (MM–PBSA) and residue–residue contact score (RRCS) analyses. A full-length human T1R1/T1R3 heterodimer was constructed with AlphaFold3. MD simulations indicated that the binding of FR-9 led to a large structural fluctuation, a large radius of gyration, and a large solvent accessible surface; on the contrary, FE-5 yielded the most stable receptor–ligand complex. The MM-PBSA analysis showed that the binding free energies of the three peptides were in the order of FR-9 > EK-5 > FE-5. The RRCS analysis indicated that RRCS values per residue were in the order of FR-9 < EK-5 < FE-5, in line with the reported umami score, and that the highest taste score of FE-5 originated from the hydrophobic interactions between Glu301 (receptor) and Phe1 (ligand) as well as the salt bridges between arginine (Arg277 and Arg307, receptor) and glutamic acid (Glu4 and Glu5, ligand) residues. These findings show that structural stability and residue contact density were more informative than binding affinity for distinguishing the taste intensity of umami peptides.

## 1. Introduction

Umami, a basic taste modality, is mediated by a variety of taste receptors. They are reported to belong to class C of G protein-coupled receptors (GPCRs) [1,2] and are generally sorted into two categories: the T1R1/T1R3 heterodimer and metabotropic glutamate receptors (mGluRs) [2,3,4]. The T1R1/T1R3 receptor is mainly located at the front of the tongue and plays a vital role in preference behavior [2,5]; it is therefore acknowledged as the main umami receptor [1,2,3,6]. T1R1/T1R3 comprises an extracellular Venus flytrap domain (VFTD), a cysteine-rich domain (CRD), and a trans-membrane domain (TRD) [1,2]. The VFTD is reported to be responsible for binding of umami ligands; then, ligand binding induces changes in receptor conformations and initiates downstream gustatory signal transduction [2,7]. Umami peptides are typically short with molecular masses less than 3 kDa [8], and they are usually isolated from protein-rich foods such as peanuts, hams, meat hydrolysates, wheat gluten, soy, and sea products [8,9,10,11,12,13,14]. Because of their ability to enhance flavor and reduce the use of salt and monosodium glutamate, umami peptides have attracted increasing interest in the food industry and taste research [8,15].

A standard workflow for the discovery of umami peptides usually comprises the three steps of identification, screening, and evaluation [16]. The experimentally identified peptides are virtually screened using prediction tools in terms of taste, activity, toxicity, and/or water solubility [16,17,18,19]. Molecular docking and machine learning approaches show great potential for a high-throughput screening [16,17,18,19,20]. Subsequently, the target peptides are synthesized with high purity for further evaluation. Umami intensity can be evaluated via a number of methods such as physical and chemical indicators, sensory evaluation, electronic tongues, and bionic taste sensors [2,21]. After peptide discovery, molecular docking is also used to investigate the interaction between taste peptides and receptors at the molecular level, enabling the prediction of binding poses, binding affinities, and driving forces for receptor–ligand interactions such as hydrogen bonding, hydrophobic interactions, and electrostatic interactions [19,20,22]. As a powerful supplement to the experiments, molecular dynamics (MD) simulation provides deep insight into the interactions between the receptor and umami peptides such as structural rearrangement, ligand binding stability, and binding energetics [23,24,25,26].

The reported umami peptides are generally short with 2–10 amino acids [27,28], and the peptide sequence length appears to affect umami intensity. A longer peptide often leads to a stronger binding affinity with the receptor, while it does not necessarily imply a stronger umami taste [29,30]. The intensity of umami peptides with four or more amino acids has been shown not to positively correlate with the peptide chain length [2]. For instance, we previously isolated three oyster-derived peptides, FLNQDEEAR (FR-9), EEFLK (EK-5), and FNKEE (FE-5). Docking predictions indicated that the nonapeptide FR-9 had a strongest binding affinity with the T1R1/T1R3 receptor, whereas it gave the lowest umami score [20]. The amino acid composition, sequence arrangement, terminal residues, and receptor-binding mode are also influential [31].

Here we construct a full-length human T1R1/T1R3 heterodimer using AlphaFold3 [32] and attempt to explore the differences in the umami scores for our previously identified peptides [20], FR-9, EK-5, and FE-5, via the computational approaches of molecular docking and MD simulations, as well as molecular mechanics Poisson–Boltzmann surface area (MM–PBSA) and residue–residue contact score (RRCS) analyses. This work provides preliminary insights into potential receptor–peptide interactions and helpful implications for distinguishing the taste intensity of umami peptides via a variety of computational approaches such as molecular docking and MD simulations.

## 2. Results and Discussion

### 2.1. T1R1/T1R3 Receptor Model

**Figure 1:**
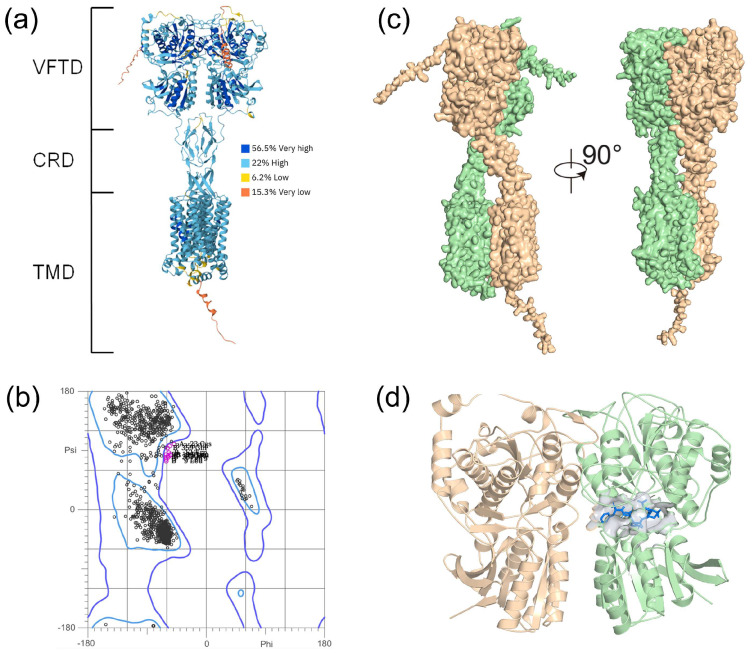
(**a**) AlphaFold3 prediction of the full-length T1R1/T1R3 receptor structure colored by pLDDT (predicted Local Distance Difference Test) confidence scores. A full-length T1R1/T1R3 receptor comprises three domains: VFTD, CRD, and TMD. (**b**) Ramachandran plot of the receptor model. The inner “favored” contour and the outer “allowed” contour were colored in sky blue and violet, respectively. The residues in “disallowed” regions were marked in magenta. (**c**) Complexation of T1R1 (colored in orange, chain A) and T1R3 (green, chain B). (**d**) Binding of umami peptide FE-5 (colored in blue) in the VFT domain. The VFTD was used for molecular docking and MD simulations.

A full-length complex model of taste receptor type 1 member 1 (T1R1) and taste receptor type 1 member 3 (T1R3) was predicted with AlphaFold3, where both receptors formed a heterodimer composed of a VFT domain (VFTD), a cysteine-rich domain (CRD), and a trans-membrane domain (TMD). A large portion of the T1R1/T1R3 complex was modeled with high confidence, as indicated by a high pLDDT (predicted Local Distance Difference Test) value of >70 (colored in blue, Figure 1a), whereas the prediction appeared to fail for T1R1 residues 1–27 and 348–360 and for T1R3 residues 1–21 and 827–852, as indicated by the model colored in yellow and orange (Figure 1a). The stereo-chemical quality of the receptor model was then evaluated by a Ramachandran plot, where 97.5% (1647/1689) of the residues were located in favored regions and 99.3% (1677/1689) were located in allowed regions (Figure 1b). These disallowed residues were mainly positioned outside the VFTD pocket and were therefore unlikely to affect subsequent ligand binding analysis. The complexation mode of T1R1 and T1R3 is shown in Figure 1c. The VFTD was responsible for the recognition of umami ligands [7,33], and was therefore chosen for subsequent molecular docking and MD simulations (T1R1: residues 22–487; T1R3: residues 23–489). The binding of peptide FE-5 within the VFTD is illustrated in Figure 1d.

### 2.2. Binding Poses of Umami Peptides with T1R1/T1R3

**Figure 2:**
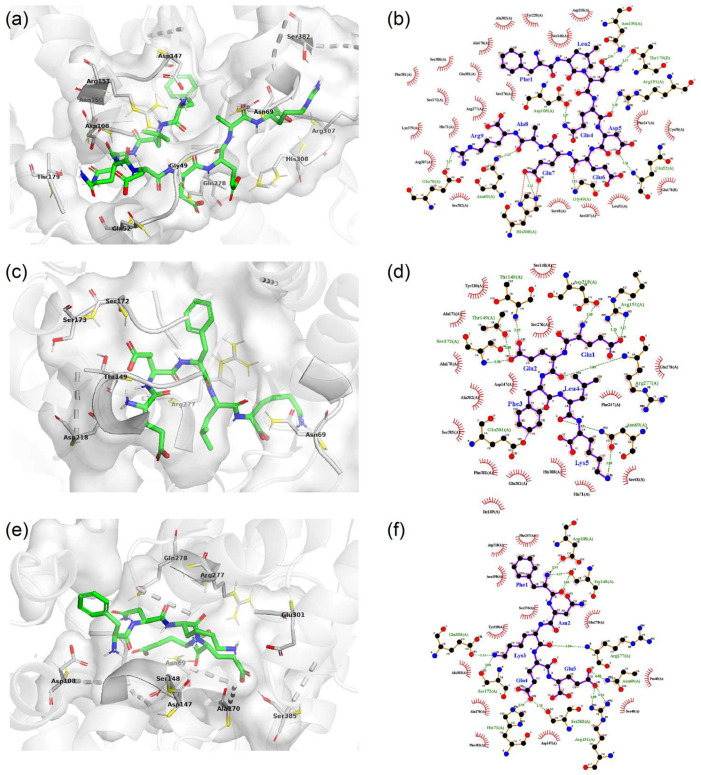
Binding poses of umami peptides in the VFTD of the T1R1/T1R3 receptor predicted by AutoDock Vina 1.2.6 (**a**,**b**) for FR-9; (**c**,**d**) for EK-5; (**e**,**f**) for FE-5. The 3D binding poses (**left**) were made using the PyMOL software (version 3.1), and the residues involved in hydrogen bond interactions were labeled. The 2D interaction diagrams (**right**) were made with the LigPlot+ software (version 2.2). The receptor residues with nonbonded contacts with the peptide are shown with spoked arcs and the receptor–ligand hydrogen bonding interactions are represented by green dotted lines.

The binding poses of the three umami peptides were predicted by molecular docking with Autodock Vina [34] and by AlphaFold3 [32]. The Vina docking results (3D and 2D binding modes) were presented with hydrogen bonds (green dotted lines) and nonbonded contacts (red spoked arcs) between receptor and ligand (Figure 2). Vina scoring predicted binding affinities of −10.1, −9.5, and −9.5 kcal/mol for FR-9, EK-5, and FE-5, respectively (Table 1). FR-9 showed the strongest binding strength with T1R1/T1R3, which can be ascribed to the fact that its long sequence length made more contacts with the receptor than EK-5 and FE-5.

For a detailed description of receptor–ligand interactions, the docked poses were analyzed by the PLIP (version 2.4.0) software [35] (https://plip-tool.biotec.tu-dresden.de; accessed on 28 April 2026), as summarized in Table 1. Four main types of interaction were identified, namely, hydrogen bonds, hydrophobic interactions, salt bridges, and π-stacking. For all three umami peptides, Asn69 was able to form hydrogen bonding interactions, and Tyr220, Gln278, and Ala302 offered hydrophobic contacts.

**Figure 3:**
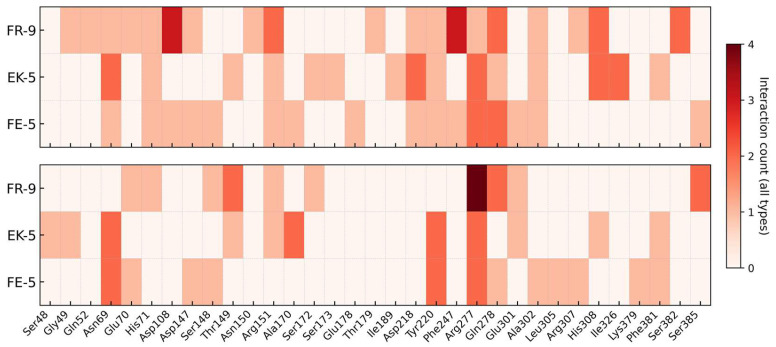
The number of interactions per residue in the T1R1/T1R3 receptor upon binding to three umami peptides for the predicted receptor–ligand complexes via Autodock Vina (**top**) and AlphaFold3 (**bottom**).

The complexes of T1R1/T1R3 with the three umami peptides were also predicted by AlphaFold3. FR-9 and FE-5 appeared to give a shallower penetration into the binding pocket of the VFT domain compared to the Autodock Vina predictions (Figure S1 in the Supplementary Materials), and the binding energies were −1.2 and −1.1 kcal/mol, respectively (Table S1). EK-5 showed a positive binding energy of 3.6 kcal/mol, implying the existence of unfavorable interactions evaluated by the Vina score function [34]. The counts for receptor–ligand binding poses for both methods are presented in Figure 3, Table S2, and Figure S2. The Vina docking tended to yield more interactions than AlphaFold3 (Figure 3 and Figure S2). The hot residues were found to be Asn69, Arg277, and Gln278 with multiple interactions with the umami peptides. For instance, Arg277 formed a hydrogen bond with Lys3 and a salt bridge with Glu5 of the FE-5 peptide for the Vina prediction.

### 2.3. Molecular Dynamics Simulations of Receptor–Peptide Complexes

**Figure 4:**
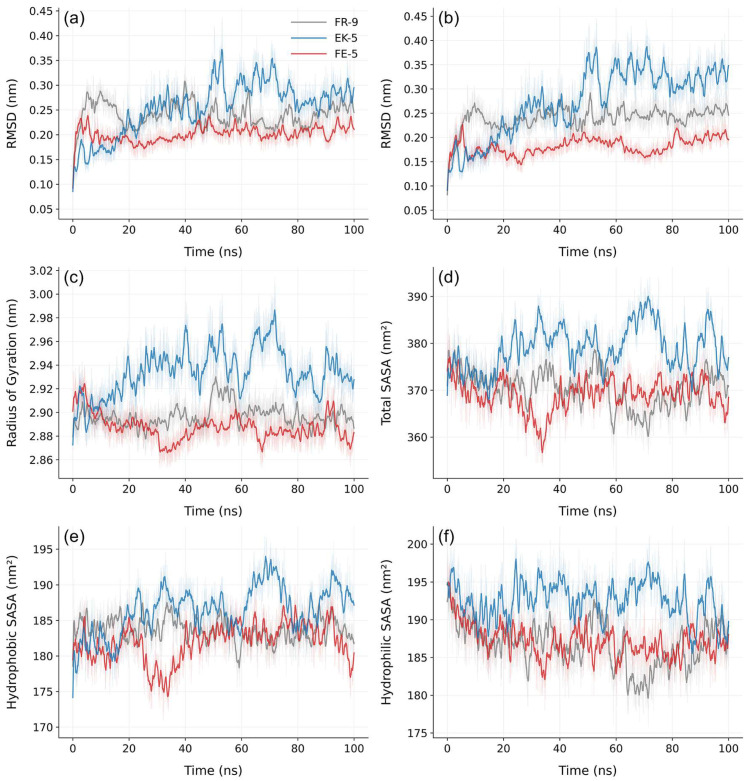
Root-mean-square deviation (RMSD) of protein backbone of T1R1/T1R3 complexes (**a**) and T1R1 complexes with umami peptides (**b**) after least-squares fitting of the structures on top of the T1R1/T1R3 receptor as well as the radius of gyration (**c**), total solvent accessible surface area (SASA, (**d**)), hydrophobic SASA (**e**), and hydrophilic SASA (**f**) for the receptor–ligand complexes as a function of simulation time. The raw data were smoothed using a sliding window of 625 (**a**–**c**) or 25 (**d**–**f**) data points.

Using the Vina docking poses as initial configurations, we performed 100 ns MD simulations to evaluate the structural stability of receptor–ligand complexes. After 70 ns simulations, the root-mean-square deviation (RMSD) of the protein backbone of the T1R1/T1R3 receptor (Figure 4a) and T1R1–peptides (Figure 4b) appeared to converge (Figure 4a); that is, the receptor or receptor–ligand complexes were sampled with a population of configurations with significant statistical weights. The binding of EK-5 led to a large structural fluctuation of the receptor with an RMSD of 0.27 nm (Figure 4a) and receptor–ligand complexes with an RMSD of 0.32 nm (Figure 4b), while FE-5 gave rise to the most stable receptor–ligand complexes, as indicated by a small RMSD of 0.20 nm (Figure 4b).

The global compactness of the receptor–ligand complexes was evaluated by the radius of gyration (*R*_g_). *R*_g_ is a rough prediction for the radius of the simulated solute assuming that the molecule of interest is spherical. As shown in Figure 4c, EK-5 gave a large *R*_g_ of 2.93 ± 0.01 nm, while FE-5 and FR-9 showed a relatively small *R*_g_ of 2.89 ± 0.01 nm. This indicated that FE-5 and FR-9 might promote a more compact receptor–ligand conformation, which corresponded to a smaller total solvent accessibility surface area (SASA) compared to the case of EK-5 (Figure 4d). A less compact receptor would allow more contacts with water molecules, thus resulting in larger values for both hydrophobic (Figure 4e) and hydrophilic (Figure 4f) SASA. Similar findings were observed for *R*_g_ and the SASA of EK-5 and FE-5 (Figure 4c–f). The umami scores were in the order of FE-5 > EK-5 > FR-9 [20]. MD simulations indicated that the high umami score of FE-5 may result from the stable receptor–ligand complexes.

### 2.4. MM-PBSA Analysis

MM-PBSA analysis was performed to further characterize the energetics of peptide binding, and the energy components are tabulated in Table 2. The van der Waals (Δ*E*_vdW_), electrostatic (Δ*E*_elec_), and nonpolar (Δ*G*_nonpolar_) interactions favored the complex formation for all three peptides, whereas the polar (Δ*G*_polar_) interaction showed a large unfavorable contribution. The solvation contribution (Δ*G*_sol_) was the energy penalty to strip the solvation shell from the receptor and/or ligands, that is, the desolvation of the binding partners upon complexation. As expected, Δ*G*_sol_ was positive and unfavorable. Peptide binding resulted in an entropy loss (negative Δ*S*) and 60–70% of the enthalpy gain (Δ*E*_MM_ +Δ*G*_sol_) upon complexation was canceled out via the entropy loss (−*T*Δ*S*). Compared to EK-5 and FE-5, FR-9 displayed a larger entropy loss because its long chain gave rise to a larger change in the vibrational entropy (Table S3 in the Supplementary Materials).

FR-9 displayed the strongest binding with Δ*G*_bind_ = −32.5 ± 1.1 kcal/mol, followed by EK-5 (−18.1 ± 3.2 kcal/mol) and FE-5 (−12.5 ± 1.6 kcal/mol). This order disagreed with the umami score of FE-5 > EK-5 > FR-9 [20], implying that the stronger binding did not necessarily give a higher umami score. A weak binding might allow a flexible movement of ligand within the VFTD binding pocket and hence more contacts with the receptor.

**Figure 5:**
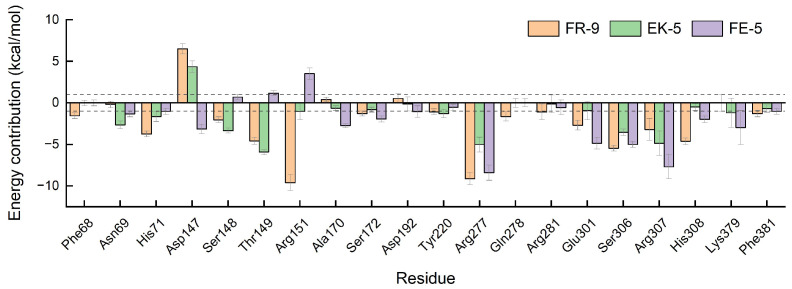
Energy contribution per residue to the binding of umami peptides with the receptor computed by the MM-PBSA analysis. Residues with contributions of ≥1 kcal/mol (as indicated by dashed lines) for at least one residue are shown here.

The total binding energies were further decomposed into contributions from each residue to pinpoint and identify key residues (Figure 5). His71, Arg151, Arg277, and Ser306 showed favorable interactions with FR-9, whereas Asp147 disfavored the binding. For EK-5, Asn69, Ser148, Thr149, Arg277, Ser306, and Arg307 provided a large contribution favoring the binding, while Asp147 showed the opposite. The higher umami score of FE-5 may be related with large contributions from Arg277, Glu301, Ser306, and Arg307 in the receptor. The identified key residues were in line with the previously reported ones of His71, Arg277, and Glu301 based on the experimental determination [36,37,38].

### 2.5. Residue–Residue Contact Score (RRCS)

To characterize the residue-level interaction strength, residue–residue contact score (RRCS) analysis was performed using the last 10 ns of MD trajectories. RRCS is a distance-based descriptor that quantified the strength and persistence of residue contacts over time [39]. Unlike global descriptors such as RMSD, *R*_g_, and SASA, RRCS measures how consistently specific residue pairs remained in contact over time. Therefore, RRCS was used here to compare contact profiles of FR-9, EK-5, and FE-5 and to evaluate dynamic residue contacts. A larger RRCS value indicates a more frequent contact between receptor and ligand during MD simulations.

**Figure 6:**
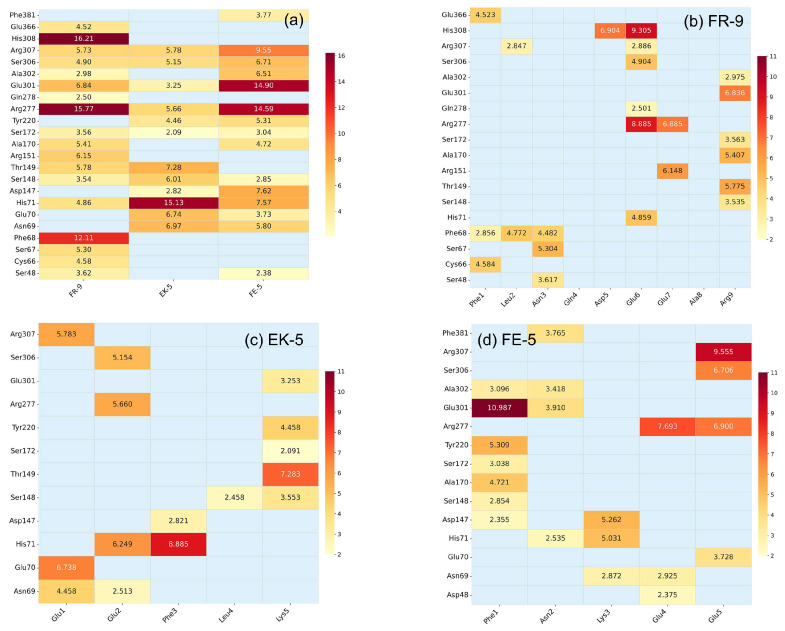
Heat maps for RRCS profiles per peptide (**a**) and per residue (**b**–**d**) of the three umami peptides, FR-9 (**b**), EK-5 (**c**), and FE-5 (**d**), with the receptor during the last 10 ns of MD simulations.

We first identified common interaction hotspots per peptide, as shown in the heat map (Figure 6a). Arg277 was a recurrent hotspot for all three peptides with RRCS values of 15.8, 5.7, and 14.6 for FR-9, EK-5, and FE-5, respectively. This indicated that Arg277 served as a crucial anchoring point in the pocket. His71, Ser306, and Arg307 also interacted with the three peptides with RRCS values of 5–15 (Figure 6a), and His71 gave the highest RRCS of 15.1 for EK-5. A large RRCS of 14.9 implied that Glu301 played a vital role in the interaction with FE-5 (Figure 6a).

**Figure 7:**
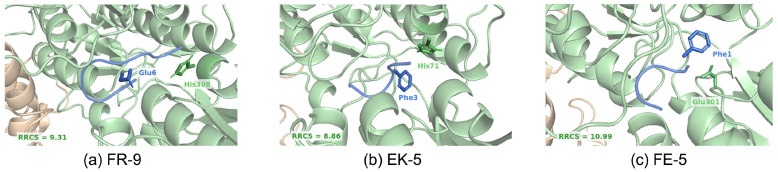
Representative receptor–ligand contact pairs for the highest RRCS value for FR-9 (**a**), EK-5 (**b**), and FE-5 (**c**). Receptor residues are shown with a stick model in green, while ligand peptides are marked in blue.

For a detailed description of contact residue pairs between receptor and ligand, interaction hotspots per residue for the three umami peptides are given in Figure 6b–d. For FR-9, the highest-scoring contact was His308–Glu6 (RRCS = 9.31), where they formed a hydrogen bond (Figure 6b and Figure 7a). For EK-5, the highest-scoring contact was His71–Phe3 (RRCS = 8.86), which was an aromatic contact (Figure 6c and Figure 7b). The highest-scoring contact for FE-5 binding was Glu301–Phe1 (RRCS =10.99), corresponding to a hydrophobic interaction (Figure 6d and Figure 7c).

FR-9 displayed a multisite interaction pattern (Figure 6b). The central Glu6 and Glu7 residues interacted with Arg277 and His308 in the pocket core, whereas the N-terminal (Phe1, Leu2, and Asn3) contacted residues Cys66, Ser67, and Phe68. At the C-terminus, Arg9 interacted with Ala302 at the rim of the binding pocket. It appeared that Gln4 and Ala8 were not involved in the receptor–ligand contacts (Figure 6b).

Unlike FR-9, each residue in EK-5 and FE-5 contributed to the binding with the receptor (Figure 6c,d) Most of the RRCS values for EK-5 were smaller compared to FE-5, implying a weaker binding of EK-5 with receptor residues His71, Ser148, Thr149, Arg277, and Arg307 (Figure 6c). FE-5 formed a dense and continuous contact network in the pocket (Figure 6d). At the N-terminus, Phe1 formed a strong contact with Glu301 and moderate contacts with Tyr220 and Ser172. At the C-terminus, Glu4 and Glu5 formed multipoint contacts with Arg277 and Arg307. These findings indicate that Phe, Glu, Lys, and Arg residues were of vital importance and responsible for the umami taste.

Table 3 summarizes the RRCS values of the three complexes. FR-9 had the highest total RRCS per peptide (114.35), whereas FE-5 had the highest RRCS per residue (19.81). EK-5 had the lowest total RRCS (71.36), while FR-9 had the lowest RRCS per residue (12.71). The RRCS values per residue were ranked in the order of FE-5 > EK-5 > FR-9, in line with the umami score [20]. This indicated that the number of contacts per residue was more informative than the total number of contacts per peptide. These residue-level differences helped explain why FE-5 showed the highest umami scores.

## 3. Materials and Methods

### 3.1. Receptor Structure Prediction

Protein sequences of human T1R1 (UniProtKB: Q7RTX1) and T1R3 (UniProtKB: Q7RTX0) were retrieved from the UniProtKB database (https://www.uniprot.org; accessed on 28 April 2026) [40]. A full-length T1R1/T1R3 heterodimer model was generated with AlphaFold3 [32], and structural quality was evaluated with MolProbity 4.5.2 (https://molprobity.biochem.duke.edu; accessed on 28 April 2026) [41].

### 3.2. Molecular Docking

#### 3.2.1. Ligand Preparation

Structural configurations of the three umami peptides FR-9, EK-5, and FE-5 were modeled with a web server of PEP-FOLD3 [42] (https://mobyle.rpbs.univ-paris-diderot.fr/cgi-bin/portal.py#forms::PEP-FOLD3; accessed on 28 April 2026) and were saved as PDB files. The MGLTools package (version 1.5.6) was used to generate the PDBQT files of receptor and ligand [43].

#### 3.2.2. Docking Parameters

Flexible docking was performed with AutoDock Vina (version 1.2.6) [34]. The center of search space 70 × 70 × 70 Å^3^ was set to (6.233, 26.492, −29.290) Å. Receptor side chains within 5 Å of the binding site were treated as flexible. The position of binding sites was referred to the predicted receptor–ligand complexes using AlphaFold3 [32]. The ligand-specific flexible residues were as follows: FR-9: Ser48, Asn69, Thr149, Ser172, Ser276, Gln278, Glu285, Glu301, Ser306, His308, and Ser385; EK-5: Ser48, Thr149, Arg151, Glu301, and Ser306; FE-5: Asn69, Gln278, and Ser382. Ten poses were generated for each peptide, and the lowest-energy pose was selected for subsequent analysis and MD simulations. The 3D description of receptor–ligand interactions was made using the PyMOL software (version 3.1) [44], and 2D interaction diagrams were generated with LigPlot+ (version 2.2) [45]. The PLIP software (version 2.4.0) was used for the detailed detection of interaction types [35].

### 3.3. Molecular Dynamics Simulations

The receptor (VFT domain of T1R1/T1R3; T1R1: residues 22–487; T1R3: residues 23–489) and ligand (umami peptides) were modeled with the AMBER ff14SB force field [46], and water molecules were described with the TIP3P model [47]. Each system was solvated in a truncated octahedral box with a minimum buffer of 1.0 nm from the box boundary, was filled with water, and was then neutralized with sodium and chloride ions. The resulting systems contained about 29,000 water molecules. Energy minimization was performed using the steepest-descent algorithm, followed by 1 ns NVT and 1 ns NPT equilibration. Production simulations were performed for 100 ns under NPT using the GROMACS software (version 2018) at 300 K [48]. The detailed parameter settings have been reported in our previous reports [49,50,51].

### 3.4. MM–PBSA Analysis

The last 20 ns of each MD trajectories were used to compute binding free energies (∆*G*_bind_) of receptor–ligand complexes using the molecular mechanics Poisson–Boltzmann surface area (MM–PBSA) method with the gmx_MMPBSA toolkit (version 1.6.4) [52]. Before calculations, water molecules and ions were stripped from MD trajectories, and periodic boundary conditions (PBC) were treated properly. ∆*G*_bind_ was decomposed into three parts, namely, molecular mechanics (MM, ∆*E*_MM_), solvation (∆*G*_sol_), and entropy (∆*S*) contributions (Equation (1)).

(1) $${∆G}_{bind}={∆E}_{MM}+{∆G}_{sol}-T∆S={∆E}_{elec}{+∆E}_{vdW}+{∆G}_{polar}{+∆G}_{nonpolar}-T∆S$$

∆*E*_MM_ contained bonded (∆*E*_bonded_) and nonbonded (∆*E*_nonbonded_) interactions; ∆*E*_bonded_ was zero because we used a single trajectory for free and bound states of the binding partners.

∆*E*_nonbonded_ amounted to a sum of van der Waals (∆*E*_vdW_) and electrostatic (∆*E*_elec_) interactions, and for classical force fields (as did in this work), it was computed via Equation (2).

(2) $${∆E}_{nonbonded}=\sum_{i}\sum_{j,i\neq j}\left(\varepsilon_{ij}\left({\left(\frac{R_{min,ij}}{r_{ij}}\right)}^{12}-2{\left(\frac{R_{min,ij}}{r_{ij}}\right)}^{6}\right)+\frac{1}{4\pi \varepsilon_{0}}\frac{Q_{i}Q_{j}}{r_{ij}}\right)\;$$

where *ε*_ij_ and *R*_min_ are the Lennard-Jones (LJ) well depth and atom-pair distance ($r_{ij}$) at which the interacting LJ 12-6 potential reaches its minimum. *ε*_0_ is the vacuum permittivity; $Q_{i}\;$and $Q_{j}$ are atomic charges of atoms *i* and *j*.

∆*G*_sol_ can be further decomposed into polar (∆*G*_polar_) and nonpolar (∆*G*_nonpolar_) ones. ∆*G*_polar_ is the polar contribution of the surrounding solvent and is treated in an average fashion via solving the Poisson–Boltzmann (PB) Equation. ∆*G*_nonpolar_ is proportional to the total solvent accessible surface area (SASA) of the molecules of interest with a proportionality constant [52,53].

The entropy was evaluated using the normal mode (NMODE) module of gmx_MMPBSA (version 1.6.4) [52], and it can be decomposed into three terms, namely, translational, rotational, and vibrational contributions (Equation (3)).

(3) $$∆S={∆S}_{trans}+{∆S}_{rot}+{∆S}_{vib}$$

### 3.5. RRCS Analysis

Residue–residue contact scores were computed using the last 10 ns of MD trajectories by the gmx_RRCS tool (version 1.0.5) with the parameters of *d*_min_ = 3.23 Å and *d*_max_ = 4.63 Å [39]. Contacts were counted whenever any atom pair fell within this distance, accumulated over the trajectory, normalized to residue–residue scores, and visualized as heat maps.

## 4. Conclusions

Using the AlphaFold3 model of a full-length human T1R1/T1R3 receptor, we combined molecular docking, molecular dynamics simulation, molecular mechanics Poisson–Boltzmann surface area (MM-PBSA) analysis, and residue–residue contact score (RRCS) analysis to investigate the molecular mechanism of the binding of oyster-derived umami peptides with the T1R1/T1R3 receptor. The experimental umami score of the three peptides was in the order of FE-5 > EK-5 > FR-9 via sensory evaluation [20]. In general, a strong binding strength with receptor is indicative of potential peptides with a high umami taste. The Vina docking predicted a binding strength of FE-5 = EK-5 < FR-9, while the MM-PBSA analysis yielded a prediction of FE-5 < EK-5 < FR-9. Both methods are extensively used in the screening and discovery of novel umami peptides; moreover, MM-GBSA (molecular mechanics generalized Born surface area) with a low computational cost is often used in high-throughput screening too. These results indicate that binding energies predicted by the Vina scoring and MM-PBSA analysis were not always helpful to distinguish the differences in the intensity of umami peptides. FR-9 showed the strongest binding strength with the T1R1/T1R3 receptor, likely due to its long sequence length offering more contacts with the receptor.

Our results indicated that dynamic behavior such as structural fluctuation and residue contact extracted from MD simulations might be helpful to distinguish the differences in umami intensity. The RRCS analysis shows that the Phe, Glu, Lys, and Arg residues of umami peptides were of vital importance and responsible for the binding (contact) with the T1R1/T1R3 receptor. It should be noted that the causality between residue contacts and perceived umami intensity requires further validation.

## Figures and Tables

**Table 1:** Binding energies (Δ*E*, kcal/mol) predicted by AutoDock Vina and interaction profiles of the three umami peptides with T1R1/T1R3 identified by PLIP.

Peptide	Δ*E*	Hydrogen Bonds	Hydrophobic Interactions	Salt Bridges	π-Stacking
FR-9	−10.1	G49, Q52, N69, D108, D147, N150, R151, T179(chain B), Q278, R307, H308, S382	H71, D218, Y220, F247, Q278, A302	E70, R277, H308	
EK-5	−9.5	N69, T149, S172, S173, D218, R277	H71, I189, Y220, R277, Q278, A302, H308, I326	R151, H308	F381
FE-5	−9.5	N69, D108, D147, S148, A170, R277, Q278, E301, S385	E178(chain B), D218, Y220, Q278, A302	H71, R151, R277	F247

The interacting residues are located in T1R1 (chain A in Figure 1) unless noted otherwise. Chain B indicates the residues in T1R3.

**Table 2:** MM-PBSA energy components (kcal/mol) for T1R1/T1R3 complexes with FR-9, EK-5, and FE-5 calculated using the last 20 ns of the MD trajectories.

Peptide	*q*	Δ*E*_vdW_	Δ*E*_elec_	Δ*E*_MM_	Δ*G*_polar_	Δ*G*_nonpolar_	Δ*G*_sol_	−*T*Δ*S*	Δ*G*_bind_
FR-9	−2	−70.6 ± 0.5	−615.0 ± 9.3	−685.6 ± 9.5	620.7 ± 10.3	−9.4 ± 0.2	611.4 ± 10.2	41.7 ± 0.6	−32.5 ± 1.1
EK-5	−1	−44.9 ± 0.9	−457.5 ± 8.6	−502.4 ± 8.6	458.1 ± 6.6	−6.6 ± 0.0	451.5 ± 6.6	32.9 ± 2.9	−18.1 ± 3.9
FE-5	−1	−58.1 ± 0.6	−607.1 ± 8.1	−665.2 ± 7.8	629.8 ± 6.6	−7.0 ± 0.0	622.8 ± 6.6	29.9 ± 0.2	−12.5 ± 1.6

*q* is the net charge (*e*) of peptide. Refer to Equation (1) for energy decomposition. Errors were computed via block averaging (4 ns for each block).

**Table 3:** Residue–residue contact score (RRCS) of umami peptides bound to the T1R1/T1R3 receptor.

	FR-9	EK-5	FE-5
RRCS	114.35	71.36	99.04
RRCS per residue	12.71	14.27	19.81
residues with the highest RRCS (receptor)	His308	His71	Glu301
residues with the highest RRCS (ligand)	Glu6	Phe3	Phe1
umami threshold (mg/mL)	0.58	0.55	0.38

The umami threshold was experimentally obtained via sensory evaluation [20]. A smaller threshold corresponds to a higher umami taste (i.e., a larger umami score).

## Data Availability

The original contributions presented in this study are included in the article/Supplementary Material. Further inquiries can be directed to the corresponding author.

## Supplementary Materials

The following supporting information can be downloaded at: https://www.mdpi.com/article/10.3390/molecules31122125/s1, Binding energies and interaction profiles for AlphaFold3 prediction (Table S1), comparison between Autodock Vina and AlphaFold3 predictions (Table S2 and Figures S1 and S2), and entropy decomposition (Table S3).
