# Supplementary material for: Exploring the Mechanism of Umami Peptide Binding with the T1R1/T1R3 Receptor via Molecular Dynamics Simulations
Source: Molecules. 2026 Jun 16;31(12):2125. doi: 10.3390/molecules31122125 (PMC13304789; doi:10.3390/molecules31122125)
Supplement: Supplementary file 1 [file molecules-31-02125-s001.zip › molecules-4322076-supplementary.pdf]

Supplementary Materials for

Exploring the Mechanism of Umami Peptide Binding with the T1R1/T1R3 Receptor via Molecular Dynamics Simulations

Chenyi Lu [1], Binghan Wu [1], Xianbing Xu [2] and Haiyang Zhang [1,*]

[1] Department of Biological Science and Engineering, School of Chemistry and Biological Engineering, University of Science and Technology Beijing, 100083 Beijing, China
[2] National Engineering Research Center of Seafood, Collaborative Innovation Center of Seafood Deep Processing, School of Food Science and Technology, Dalian Polytechnic University, 116034 Dalian, China
* Correspondence: zhanghy@ustb.edu.cn

Table S1. Binding energies (ΔE, kcal/mol) and interaction profiles of the three umami peptides with T1R1/T1R3 identified by PLIP.

peptide	ΔE	Hydrogen Bonds	Hydrophobic Interactions	Salt Bridges
FR-9	-1.2	E70, S148, T149, S172, R277, Q278, S385	R277	H71, R151, R277, E301
EK-5	3.6	S48, G49, N69, T149, A170, Y220, R277	A170, Y220, F381	R151, R277, E301, H308
FE-5	-1.1	N69, E70, S148, R277, Q278, A302, L305, R307	Y220, F381	D147, R277, K379

The binding poses of receptor–ligand complexes were predicted by AlphaFold3. ΔE was computed using the Vina scoring. The interacting residues were located in T1R1 (chain A in Figure 1) unless noted otherwise. Chain B indicated the residues in T1R3.

Table S2. Comparison of the number of interactions for predicted receptor–ligand complexes via Autodock Vina and AlphaFold3.

peptide	Vina					AlphaFold				
	Total	Hydrogen Bonds	Hydrophobic Interactions	Salt Bridges	π-Stacking	Total	Hydrogen Bonds	Hydrophobic Interactions	Salt Bridges	π-Stacking
FR-9	27	16	8	3	0	16	11	1	4	0
EK-5	20	8	9	2	1	15	8	3	4	0
FE-5	18	9	5	3	1	15	9	3	3	0

Interaction profiles of the three umami peptides with T1R1/T1R3 were identified by PLIP. Note that one residue may be involved with more than one interactions.

Table S3. Entropy decomposition (kcal/mol) for the binding of three umami peptides to the T1R1/T1R3 receptor.

peptide	$-T\Delta S_{\text{trans}}$	$-T\Delta S_{\text{rot}}$	$-T\Delta S_{\text{vib}}$	$-T\Delta S$
FR-9	14.0 ± 0.1	13.0 ± 0.1	14.7 ± 0.6	41.7 ± 0.6
EK-5	13.5 ± 0.1	11.9 ± 0.1	7.4 ± 2.9	32.9 ± 2.9
FE-5	13.5 ± 0.1	11.8 ± 0.1	4.5 ± 0.2	29.9 ± 0.2

Refer to Equation 3 in the main text for the energy decomposition.

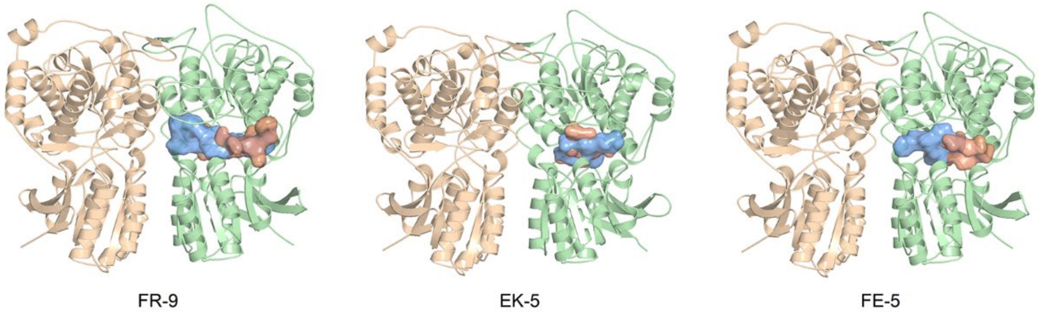

Figure S1. Comparison of the predicted receptor–ligand complexes for the three umami peptides via Autodock Vina (blue) and AlphaFold3 (brown). The VFT domain of T1R1 and T1R3 were colored in green and orange, respectively.

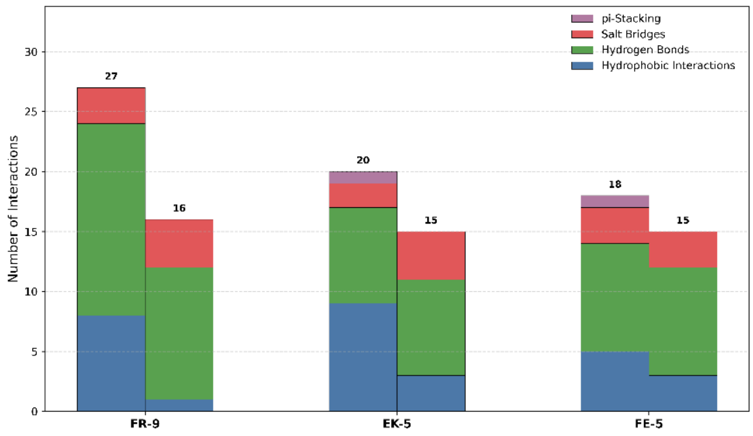

Figure S2. The number of interactions for predicated complexes between T1R1/T1R3 and three umami peptides via Autodock Vina (*left*) and AlphaFold3 (*right*). Interaction types were identified by the PLIP software (version 2.4.0).
